# The 50 Most Important Questions Relating to the Maintenance and Restoration of an Ecological Continuum in the European Alps

**DOI:** 10.1371/journal.pone.0053139

**Published:** 2013-01-14

**Authors:** Chris Walzer, Christine Kowalczyk, Jake M. Alexander, Bruno Baur, Giuseppe Bogliani, Jean-Jacques Brun, Leopold Füreder, Marie-Odile Guth, Ruedi Haller, Rolf Holderegger, Yann Kohler, Christoph Kueffer, Antonio Righetti, Reto Spaar, William J. Sutherland, Aurelia Ullrich-Schneider, Sylvie N. Vanpeene-Bruhier, Thomas Scheurer

**Affiliations:** 1 Research Institute of Wildlife Ecology, Department of Integrative Biology and Evolution, University of Veterinary Medicine, Vienna, Austria; 2 Institute of Integrative Biology, Swiss Federal Institute of Technology Zurich, Switzerland; 3 Department of Environmental Sciences, University of Basel, Basel, Switzerland; 4 Department of Earth and Environmental Sciences, University of Pavia, Pavia, Italy; 5 Unité Ecosystèmes Montagnards, National Research Institute of Science and Technology for Environment and Agriculture, Saint Martin d’Hères, France; 6 Institute of Ecology, University of Innsbruck, Innsbruck, Austria; 7 Ministry of Ecology, Sustainable Development and Energy, Paris, France; 8 Research and Geoinformation, Swiss National Park, Zernez, Switzerland; 9 Research Unit Biodiversity and Conservation Biology, Swiss Federal Institute for Forest, Snow and Landscape Research, Birmensdorf, Switzerland; 10 Task Force Protected Areas - Permanent Secretariat of the Alpine Convention, Chambery, France; 11 Partner-innen in Umweltfragen, Liebefeld, Switzerland; 12 Swiss Ornithological Institute, Sempach, Switzerland; 13 Conservation Science Group, Department of Zoology, University of Cambridge, Cambridge, United Kingdom; 14 International Commission for the Protection of the Alps-CIPRA International, Schaan, Liechtenstein; 15 Écosystèmes Méditerranéens et Risques, National Research Institute of Science and Technology for Environment and Agriculture, Aix-en-Provence, France; 16 Swiss Academy of Sciences, Berne, Switzerland; University of Bern, Switzerland

## Abstract

The European Alps harbour a unique and species-rich biodiversity, which is increasingly impacted by habitat fragmentation through land-use changes, urbanization and expanding transport infrastructure. In this study, we identified the 50 most important questions relating to the maintenance and restoration of an ecological continuum – the connectedness of ecological processes across many scales including trophic relationship and disturbance processes and hydro-ecological flows in the European Alps. We initiated and implemented a trans-national priority setting exercise, inviting 48 institutions including researchers, conservation practitioners, NGOs, policymakers and administrators from the Alpine region. The exercise was composed of an initial call for pertinent questions, a first online evaluation of the received questions and a final discussion and selection process during a joint workshop. The participating institutions generated 484 initial questions, which were condensed to the 50 most important questions by 16 workshop participants. We suggest new approaches in tackling the issue of an ecological continuum in the Alps by analysing and classifying the characteristics of the resulting questions in a non-prioritized form as well as in a visual conceptualisation of the inter-dependencies among these questions. This priority setting exercise will support research and funding institutions in channelling their capacities and resources towards questions that need to be urgently addressed in order to facilitate significant progress in biodiversity conservation in the European Alps.

## Introduction

The European Alps span eight countries, from the Mediterranean shores of Southern France to Slovenia and link with adjacent mountain ranges such as the Carpathians, Balkans and Apennines. The Alps harbour an extraordinary diversity of habitats, plants and animals including many endemics. They are considered as one of the most important regions for the preservation of biodiversity in Europe [1]. However, the Alps are also the home and workplace of up to 14 million people and a holiday destination for more than 100 million tourists each year. Anthropogenic land-use changes, urbanization and the development of transport infrastructure have fragmented the ecological continuum - the connectedness of ecological processes across many scales including trophic relationship and disturbance processes and hydro-ecological flows of the Alps [2], [3]. Plant and animal populations become increasingly isolated on ever-smaller remnant habitat patches in a human-dominated landscape. Isolation leads to increased vulnerability in the face of stochastic events and decreased genetic diversity [4]. Enhancing ecological connectivity is an integral part of the European Commission’s ambitious 2020 Biodiversity Strategy (http://ec.europa.eu/environment/nature/ecosystems/index_en.htm). During the past few years, major efforts have been undertaken to maintain and foster pan-alpine biodiversity. The “Platform Ecological Network” of the Alpine Convention (http://www.alpconv.org/en/organization/groups/WGEcologicalNetwork/default.html), the “Ecological Continuum Initiative” (http://www.alpine-ecological-network.org/about-us/ecological-continuum-initiative) and the Alpine Space project “ECONNECT” (www.econnectproject.eu) are three examples of such initiatives that also aim at enhancing ecological connectivity across the Alpine range.

ECONNECT is a project funded by the EU within the framework of the European Territorial Cooperation Alpine Space Programme and co-funded by the European Regional Development Fund. The project envisions an enduringly restored and maintained ecological continuum, consisting of inter-connected landscapes and protected areas, across the Alpine Arc region, where biodiversity will be conserved for future generations and the resilience of ecological processes will be enhanced. Employing an integrated and multidisciplinary approach, the project has provided an Alpine-wide overview on areas important for ecological connectivity by integrating quantitative and qualitative information on selected sites and the level of interconnectivity among them [5]. Additionally, social, legal and economic barriers to connectivity have been identified and proposals made on how to overcome them [6]. However, in the course of the project it became evident that relevant knowledge and information is missing despite being considered essential for on-going and future projects. This knowledge gap includes consistent scientific definitions, evidence-based assessment methods, evaluation of impacts on the ecological continuum in the Alps as well as best practice measures. Similarly, other authors have recently addressed an analogous lack of evidence in respect to the design and implementation of ecological corridors, an important conservation intervention in an ecological continuum [7]. To address and surmount this knowledge deficit, the three initiatives mentioned above formed a small core group comprising both researchers and practitioners in order to start a process identifying these knowledge gaps.

Sutherland et al. 2011 [8] introduced the method of a “priority setting exercise” to identify research needs of practitioners and policy makers. Identifying research needs is essential in furthering evidence-based conservation [9], [10]. Priority setting exercises have been successfully applied in the UK, USA, Switzerland and even on a global scale tackling such diverse issues as biodiversity, global agriculture and national environmental policy [11]–[14]. A priority setting exercise identifies existing knowledge, supports research and helps funding institutions channel their capacities and resources towards questions that need to be urgently answered in order to facilitate significant progress in a respective field: in our case biodiversity conservation in the European Alps.

In this study, we identified the 50 most important questions relating to the maintenance and restoration of an ecological continuum in the European Alps. We initiated and implemented a trans-national priority setting exercise, inviting researchers, practitioners, NGOs, policy makers and other stakeholders from the Alpine region to participate. The exercise was composed of an initial call for pertinent questions, a first online evaluation of the received questions and a final discussion and selection process during a joint workshop. Furthermore, we suggest new approaches in tackling the issue of an ecological continuum in the Alps by analysing and classifying the characteristics of the resulting questions in a non-prioritized form as well as a visual conceptualisation of the inter-dependencies among these questions.

## Results

The non-prioritized list of the 50 most important questions concerning an ecological continuum in the Alps is shown in tables 1 and 2. The resulting questions were individually classified broadly in nature-, people- and management contexts (NC, PC, MC) based on a concept previously introduced by Worboys et al. [15]. Each context area has three sub-topic areas to which the questions are finally attributed. For reasons of clarity, we assign each question to only one context area, but provide the second interrelated context and dependencies in brackets where appropriate.

**Table 1:** Non-prioritized list of the 50 most important questions.

01	Which landscape elements and land use types enhance or moderate gaps in connectivity?
02	How are corridors best implemented; with clearly spatially defined borders or as functional units integrated in wide ecological continuums?
03	How do major land use changes affect ecological connectivity across the Alps?
04	What is the relative importance of climate/land-use change to changes in the ecological continuum of Alpine regions?
05	Which indicators reflect the changes in connectivity that result from climate or human induced changes in Alpine landscapes?
06	How important is connectivity in maintaining key ecosystem services?
07	How can ecological connectivity maintain the adaptive capacity of ecosystems in the face of environmental change?
08	Which of the habitat types important for landscape connectivity are most affected by climate change
09	How does alternative energy production impact on connectivity and natural habitats?
10	What is the best method to design corridors for multiple species?
11	How severe is the current lack of connectivity between populations of alpine species?
12	What are indicators for a multi-species continuum?
13	What impacts do various seasonal leisure activities (including low-impact practices) have on ecological connectivity across the Alps?
14	How can wilderness areas (wildlife, recreation, tourism) contribute to ecological connectivity?
15	What is an effective set of indicators (i.e., for species and habitats) that can be used to evaluate and monitor ecological connectivity at different scales?
16	How does the return of large carnivores affect ecosystems in the Alpine ecological network?
17	What is the impact of gene flow through an ecological continuum on genetic adaptation to climate change?
18	How does the ecological continuum allow shifts in species distribution to keep pace with climate change?
19	Are artificially engineered ecological networks a threat or a benefit to endemic species?
20	What are the consequences for both genetic and species diversity if the system of natural barriers changes?
21	How will future changes in species distribution affect connectivity and fitness among interacting species?
22	How much gene flow fostered by connectivity is beneficial to populations and species without disrupting local adaptations?
23	How can the spread of invasive species and diseases be minimized, while ensuring connectivity for native species?
24	How do elements of the ecological network affect human welfare and perception?
25	How can agricultural and silvicultural land use be optimised in order to promote and conserve ecological connectivity?
26	How can connectivity for biodiversity and ecosystem conservation become and be managed as a public good?
27	How do demographic changes in the Alps affect the future ecological continuum?
28	How do the aims of ecological connectivity and tourism conflict?
29	What is the most effective way to employ the different categories of protected areas to ensure connectivity and the provision of ecosystem services in the Alps?
30	How can we use and integrate existing instruments and programmes to enhance trans-sectoral funding for ecological connectivity?
31	How can ecological connectivity be integrated into spatial and infrastructural planning and legislation at various administrative levels?
32	How can legal and conceptual tools stimulate the development of trans-border connectivity?
33	How is it possible to harmonise contradictory, competing spatial sectoral policies in order to enhance connectivity?
34	Which policy-measures are necessary to safeguard the ecological network beyond protected areas?
35	Which of the existing sectoral funding systems have a positive and which have a negative effect on connectivity?
36	What incentives for agriculture and forestry are needed to maintain and restore ecological connectivity in different Alpine areas?
37	Which strategy, integration or segregation, is more appropriate for promoting ecological connectivity in different alpine areas?
38	How can we effectively manage areas heavily affected by tourism in order to maintain their function within an ecological continuum?
39	How can we enhance sharing of theoretical and empirical good practice knowledge amongst and between sectors?
40	How can the management of protected areas better incorporate functional relationships with surrounding areas?
41	Which specific restoration measures can increase connectivity?
42	What kind of monitoring is needed to evaluate the long-term efficiency of connectivity measures in the face of dynamic anthropogenic change?
43	How can an alpine-wide, accessible and effective connectivity data platform be created?
44	How can databases for existing or emerging bio- and geo-data be improved for the promotion of connectivity projects in the Alps?
45	What is the effectiveness of different methods (e.g. sensor data) to monitor the consequences of structural connectivity or its elements across different spatial and temporal scales?
46	What is the effectiveness of different methods to record the effectiveness of functional connectivity or its elements across different spatial and temporal scales?
47	How can we use evidence-based education to increase public awareness of ecological networks?
48	How can methods of conflict resolution be adapted and/or used to mitigate concerns and obstruction to ecological networks?
49	How should we integrate spatial and temporal dynamics into the realization of the Alpine ecological continuum?
50	How can the species and habitat approaches to designing ecological connectivity be integrated into the process of landscape planning?

**Table 2:** Non-prioritized list of the 50 most important questions concerning an ecological continuum in the Alps classified into nature-, people- and management contexts.

No.	NATURE CONTEXT			PEOPLE CONTEXT			MANAGEMENT CONTEXT			
	Structural andfunctionalconnectivity	*Habitat* *connectivity*	*Evolutionary* *process and* *connectivity*	*Natural land: social,* *cultural and* *spiritual values*	*Life support* *needs*	Economic,social andpolitical needs	*Legislation,* *policy and* *planning needs*	*Management* *support needs*		*Tools, incentives, knowledge*
1	NC									
2	NC									MC *(depends on question 15)*
3	NC					PC				
4	NC									
5	NC									MC
6	NC				PC					
7	NC									
8		NC (depends on questions 1 and 5)								
9		NC			PC					
10		NC								MC *(depends on question 12)*
11		NC								
12		NC								X
13		NC				PC				
14		NC					MC			
15		NC								X
16		NC								
17			NC							
18			NC							
19			NC							MC (*depends on questions 11 and 15)*
20			NC							
21			NC							
22			NC							
23			NC							
24				**PC**						
25	NC				**PC**					
26					**PC**					MC
27	NC					**PC**				
28		NC				**PC**				
29	NC						**MC**			
30							**MC (** *depends on question 35)*			
31							X			
32						PC	X			
33						PC	X			
34							X			
35							X			
36							X			
37							X			
38						PC			**MC**	
39									**MC**	
40						PC			**MC**	
41	NC									**MC**
42						PC				**MC**
43										**MC**
44										**MC**
45	X									**MC**
46	NC (*depends on question 15)*									**MC**
47				PC						**MC**
48						PC				**MC**
49										**MC**
50		NC								**MC**

The largest proportion of questions (46%) was attributed to the nature context. The nature context questions are rather evenly distributed between the three subtopics. This is followed by the management context (44%) where by far the largest proportion of questions relates to the legislation, policy and planning needs subtopic (63%). Finally the people context makes up a mere 10% of the total questions. From the 50 questions, by far the largest fraction (60%) was formulated as “how” questions, followed by “what” (26%) and “which” questions (14%). Consequently, most attention was given to transformation processes aiming at practices to improve the current situation in Alpine connectivity.

### Visualization of Interactions, Inter-relations and Dependencies of the 50 Final Questions

**Figure 1:**
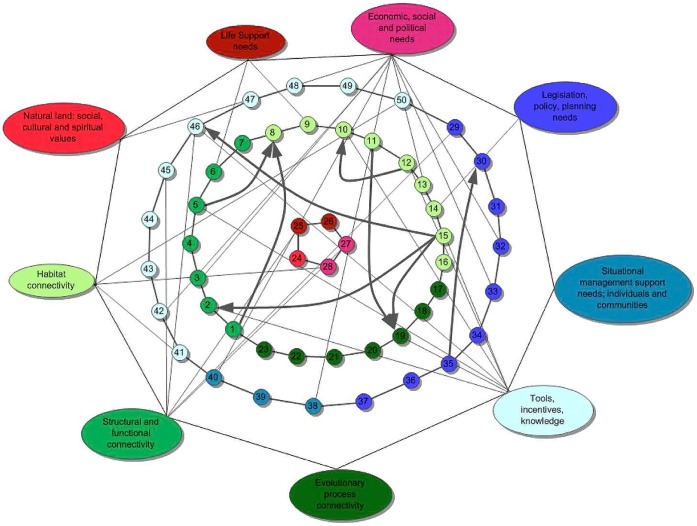
Web of the 50 most urgent questions concerning an ecological continuum in the European Alps. The nine sub-topics of the three context areas (nature: green; people: red; management: blue) mark the edges of the web. The questions of each context area are interlinked and marked in the same colour as the sub-topics they were assigned to. Questions (numbers; see text) which also address another context area are further linked with a second sub-topic, highlighting the interactions. Bold arrows indicate that one or more questions need to be solved before a subsequent question can be answered.

We created a graph in the form of a web to illustrate the “location” of each question within the framework of the nine sub-topics in the nature-, people- and management-context (Figure 1). Each context and its assigned questions are shown in the same colour, whereas the sub-topics in each context area are given in varying shades of the same colour (NC: green; PC: red; MC: blue). The sub-topics designate the edges of the web, while the questions from one context area are arranged in a connected cycle in the middle of the web to illustrate their interactions. Questions, which were also assigned to a second sub-topic of another context area, are interlinked with a grey line. Bold arrows indicate that this particular question has to be answered first before a subsequent one (to which the arrow points) can be addressed. In some cases, answers from two previous questions are required for the solution of a third question (e.g. question 8 depends on the answers from questions 1 and 5) whereas in other cases, two or three questions depend on the answer of a single question in advance (e.g. questions 2, 19 and 46 depend on the answer of question 15).

Overall, 27 questions were assigned to a second context area due to their transdisciplinary nature. The sub-topics 6 “economic, social and political needs” (PC) and 9 “tools, incentives, knowledge” (MC) showed the most inter-relations to other context areas (8 and 7 links, respectively).

## Discussion

The aim of this study was to identify and analyse gaps of knowledge with respect to achieving, restoring and maintaining an ecological continuum in the European Alps. In order to identify information gaps, we implemented an alpine wide “priority setting exercise” as previously described by Sutherland et al. [12]. This exercise was a useful and efficient tool to compile inputs from various researchers, practitioners, administrators, stakeholders and policy makers from different countries with a relatively low initial effort. The major part of the process was performed via email communication and was administered part-time by one person. We feel that this resource-saving method is a strong argument in favour of this approach, especially given the generally limited resources for connectivity conservation [16], [17].

The output of priority setting exercises depends on a number of factors, such as the selection of participants, the number of individuals effectively involved in formulating the questions and on the voting system [11]–[13]. One selection bias that could arise as a consequence of listing some 400+ questions is that an individual performing the selection process is almost certainly going to become tired as he proceeds down the list. In order to address this possible bias in future exercises, it would be prudent to provide the numbered questions in a individualized random sequence to the participants. Decisions claim to be objective, however, since people’s choices are subjective, the only option is to make them “to the best of one’s knowledge”. Only a constant review-process from the organisational core team, consisting preferably of several people from different fields, and continuous discussion with and feedback from other colleagues can minimize the inherent bias. Recently Braunisch et al. [14] have reported on a two-step evaluation of a similar priority setting exercise that elegantly addresses bias through a process of rating and speciation of the questions. The composition of the workshop participants exemplifies this problem. Although we invited six policy makers to join the initiative, only two generated initial questions and only one participated in the workshop. The problem of a non-representative group of experts missing valuable issues has been pointed out by previous authors [14], [18]). In future efforts of this type, it could be beneficial to provide more information to the potential participants in order to alleviate any concerns as to information from discussions being individually attributed and distributed outside the framework of the actual exercise. Clearly stating, and explaining if necessary, that the Chatham House Rule: “When a meeting, or part thereof, is held under the Chatham House Rule, participants are free to use the information received, but neither the identity nor the affiliation of the speaker(s), nor that of any other participant, may be revealed”. (http://www.chathamhouse.org/about-us/chathamhouserule) will be adhered to is in our view beneficial to encourage openness and the sharing of information. Furthermore, as previously stated by Wuelser et al. [19] effective transdisciplinary problem framing and research orientation exercises require a careful specification of context and purpose. Possibly, this exercise was unsatisfactorily framed with a generic “identifying knowledge gaps”. Without a clearly defined output context it can be difficult for an individual to select the most important questions from the bulk of initially proposed questions.

A further issue that hindered and complicated the exercise is the fact that the Alps are distributed across eight countries, 28 regions, 98 provinces and 5 spoken languages. Participants originated from five alpine countries (in the collection phase complemented by one participant from the UK) and divergent cultural backgrounds. In contrast to most previous studies that were English-language based, this study involved four different languages (English, German, French and Italian), which made the succinct formulation of questions particularly difficult. We tried to alleviate the fact that English was a foreign language to all of the participants by consulting with a native speaker during the entire process. Similarly, Braunisch et al. [14] had to deal with multiple languages in their study and in contrast to this study opted to translate questions into the two main local languages (French and German). Moreover, we tried to address uncertainties in the formulation of questions by making direct queries to the main author of a question if needed and encouraged all participants to do the same during the evaluation process and the workshop. It is interesting to note that numerous questions are formulated in very generic terms. While this is possibly a result of language constraints, it can, in these authors’ view also be seen as a consequence of the nonspecific use of the term “connectivity” and the wide-ranging interpretation and understanding of the term. In contrast to a previous study in Switzerland (Braunisch et al.) it is interesting to note the absence of species- specific questions and the significant integration of questions related to ecological theory (14%) in this study. While the significance of this theoretical basis has been recognized in this exercise it is clear that these questions are inherently difficult to address and implement into action [20] We analysed the interactions, inter-relations and dependencies of the final questions that resulted from the priority setting exercise in order to suggest approaches to answer these questions. Therefore, we classified the questions into a nature-, people- and management context, applying a concept previously introduced by Worboys et al. [15]. The majority of the questions (54%) could be assigned to more than one context area, indicating the transdisciplinary character of the results. It is important to note that the resulting list is neither prioritized in respect to the individual questions nor in respect to the assigned contexts. The nature context though listed first does not necessarily take precedence over the people and management contexts in framing this problem. It is interesting to note that while only 4 questions were attributed to the people context, these were highly inter-linked to other questions clearly demonstrating in this case the pivotal role of economic, social and political needs. Braunisch et al. have previously showed a similar importance of questions reconciling biodiversity conservation with societal and economic goals for Switzerland [14] though, the goal of this exercise was a non-prioritized list, eleven questions where directly dependent on each other, suggesting that a chronological order must be considered when addressing these problems. This fact inherently creates some prioritization. In order to answer question 8 “Which of the habitat types important for landscape connectivity are most affected by climate change?”, we first need to understand question 1 “Which landscape elements and land use types enhance or moderate the gaps in connectivity?”. Furthermore, the answer to question 5 “Which indicators reflect the changes in connectivity that result from climate or human induced changes in Alpine landscapes?” is also essential to adequately address question 8 above. Hence, question 8 depends on the results of two other questions.

Similarly question 15 “What is an effective set of indicators (for species and habitats) that can be used to evaluate and monitor connectivity at different scales?” is a prerequisite in addressing 3 other questions: namely question 2 “How are corridors best implemented; with clearly spatially defined borders or as functional units integrated in wide ecological continuums?”, question 19 “Are artificially engineered ecological networks a threat or benefit to endemic species?” and question 46 “What methods can be used to record the effectiveness of functional connectivity, or its elements, across different spatial and temporal scales?”. Essential questions like question 15, dealing with indicators and methodological approaches, should be given clear priority, as their answer is a precondition for scientifically sound answers to other, subsequent questions.

Solving certain questions in a consecutive order indicates a potential time lag between putting forward a question and actually answering it. This time lag may lead to a change of the initial situation, which would imply that the results of the preceding question are no longer suitable for solving the initial issue. This time lag in connectivity conservation makes it impossible to “come back” to the initial situation after the extended process of research, decision-making and implementation. This clearly indicates that there is an asymmetric drift of the initial problem and its proposed solution due to the general inertia in decision-making processes dealing with environmental issues. As holds true for numerous environmental problems, most notably global climate change, we suggest that the conservation and restoration of an ecological continuum is a “wicked problem” [21]. The example of question 19 clearly demonstrates several characteristics of a “wicked” and possibly even a “super-wicked problem” [22].

The gaps of knowledge, in conserving and restoring connectivity emphasized in this exercise make evident that these involve a highly dynamic and interconnected process rather than a simplistic and straightforward approach. It appears essential to reconcile the dynamic and complex nature of the problem with the problem solving approaches. Inadequate simplification of the interdependencies will possibly lead to results that are not relevant in forming policy [19]. Furthermore, our results indicate that maintaining and restoring ecological connectivity in the Alps is most likely a “super-wicked problem”, and this implies the need for novel approaches in addressing this issue. As has been previously suggested by other authors, we also feel strongly that the usual backward looking method of investigating the past and generating selective and singular predictions, is only sufficient for “tame problems” but inadequate for a highly dynamic and interconnected process such as ecological connectivity [21], [22].

In order to address the complex issue of an Alpine ecological continuum, it appears necessary to apply a forward reasoning approach which identifies possible future scenarios and integrates uncertainties [23]. It is somewhat surprising that questions concerning how ecological connectivity is affected and can be managed make up the largest percentage (60%) of the generated questions. Authors from the field of transdisciplinary research have termed knowledge related to this type of question “transformation knowledge” [24], [25]. These questions deal with the genesis and future development of a problem and subsequently with the interpretation and perception of the problem in the “real world”. “What” questions address determining factors of connectivity, and answers to such questions provide “system knowledge”. Finally, “which” questions address desired goals and better practices. This has been termed “target knowledge”. Each of these knowledge forms has specific challenges and notably “system knowledge” must confront uncertainties. It is essential to understand that solutions are only possible when the other postulated forms of knowledge, “target-” and “transformation knowledge”, are integrated into the solution-mix. Another method introduced by Sutherland et al. [26], [27], called “horizon scanning”, an exercise that focuses on potential threats and opportunities in the future rather than identifying present knowledge gaps, may supplement and enhance this approach.

The visual “chaos” and multi-structural character of our results, shown in the “web of questions” (Figure 1)**,** reflects the sectoral structure of society, governance and administration with respect to environmental problems in general. To overcome this, an integrative transdisciplinary approach is necessary. What appears to be missing in order to find a starting point to address the problem of the Alpine ecological continuum, is a common strategy or vision [5], [15]. In the authors’ view, this is also supported by the fact that the largest percentage of the formulated questions questioned the manner, condition or quality of ecological connectivity. This exemplifies the necessity of generating “system knowledge” and confronting uncertainties. Total conformity among all actors in the search for a common denominator is unrealistic and cannot be a realistic goal, as Worboys et al. [15] have pointed out, but a clear vision that “expresses the joint aspirations of leaders, managers and participants in the initiative, without closing off avenues for constructive debate and disputation” to support and sustain connectivity conservation may be a starting point. Possibly, ecological connectivity can constitute a common “anchor” for trans-sectoral deliberations on biodiversity conservation. However, in order to not become overburdened by the complexity of the issue, it appears essential to address the inherent complexity within a well-reflected investigational framework [19].

For this type of study to provide guidance and contribute towards conservation-action implementation the results must be disseminated accordingly. As has been pointed out previously, bridging the gap of knowledge between research and conservation practice cannot be achieved with unidirectional platforms [28], [29]. While other authors have suggested that new platforms of bidirectional knowledge dissemination must be developed these authors believe that it is more efficient to employ and if necessary adapt existing information platforms. The results from this study will be disseminated over existing platforms, most importantly the “Platform of Ecological Networks” of the Alpine Convention. This platform encompasses representatives of the Alpine countries, protected areas, Alpine institutions and experts. It strives to provide a bidirectional link between policy makers, the scientific community and practitioners and encourages more efficient cooperation with other sectors [30]. As has been pointed out by Braunisch et al. [14], integrating scientifically trained and transdisciplinary-cognizant personnel that can translate science and ease the exchange of information could significantly enhance the effectiveness of such platforms. In these authors view, an initial task of the platform could be to organize and facilitate research and conservation-action activities centred on the inter-dependent questions identified in this study. It is the authors’ opinion that this priority setting exercise and the subsequent dissemination of results will support research and funding institutions in channelling their capacities and resources towards questions that need to be urgently addressed in order to facilitate significant progress in biodiversity conservation in Europe and specifically in the Alps. Furthermore, the definition of 50 most important questions is an important first step towards a common and harmonized approach in maintaining and enhancing ecological connectivity across the heterogeneous Alpine arch.

## Methods

### Priority Setting Exercise to Identify Gaps of Relevant Knowledge and Generating the most Urgent Questions

A core team of five researchers and practitioners initiated and facilitated the entire exercise. The priority setting process was divided into four steps: initiation, assembling and organizing initial questions, pre-workshop voting and workshop voting. The method has been previously described in detail in various publications [11]–[13]. Therefore, we only briefly outline the main steps of the process. Further information on characteristics of the participants and the voting process are given in Table 3.

**Table 3:** Characteristics and voting systems of the priority setting exercise.

Pre-workshop				Workshop		
Participatingcountries	Characteristicsof participatingorganisations	Number ofparticipatingorganisations^1^/number ofindividualsinvolved ingenerating initialquestions	12 main topics towhich initial 484questions wereassigned to	Number andcharacteristics ofworkshop participants(organisations/individuals)^1^	Pre-workshopvoting system	Workshop votingsystem
Austria, Germany,France, Italy,Liechtenstein,Switzerland	Researcher(n = 12),practitioners(n = 6), NGOs (n = 4), policymakers (n = 2),network (n = 1)	26 organisations/109 individualsgenerating 484initial questions	Climate change,protected areas,indicators, speciesbiology, naturalnetworks andbarriers, spatialdevelopment andlegal constraints,monitoring and datamanagement, habitatmanagement andland use, participationand communication,tourism and recreation,economics and ecosystemservices, multidisciplinaryapproaches and commonstrategy	14 organisationsresearch (n = 8),practitioners (n = 4),NGOs (n = 1), policymaker (n = 1)/16individuals	Votes perparticipant: 55/484× 100% questionsper topic, resultingin 385 questionsplus 15 addedquestions	Voting system forsessions: 50/400×100%questions per topic astop priority, 2 questionsper topic as secondpriority. Votingsystem for plenarysession: 50 top priorityminus the questions(dismissed in discussion)plus top ranked secondpriority questions, resultingin 50 final questions

1 including core team.

1. Initiation. Based on the individual networks of the core team and striving to depict a representative cross-section of the main players in Alpine biodiversity conservation, we invited 48 institutions including researchers, conservation practitioners, NGOs, policymakers and administrators from the Alpine area to participate in this initiative.
2. Collating of initial questions. 25 of the invited 48 institutions (52%) generated 484 initial questions. The core team collated and assigned these questions to twelve main topics for clarity (Table 3).
3. Pre-workshop voting. In order to reduce the set of questions, we distributed the initial-questions-list to all participants for a first evaluation. Rephrasing and adding of questions was actively encouraged at this stage. In preparation for the workshop phase, participants were then asked to select a maximum of 55 questions from the total of 484 questions submitted. Participants were also asked to only select questions from areas in which they had sufficient knowledge base. A total of 385 questions received at least one vote and an additional 15 questions were proposed at this stage.
4. Workshop. Final discussion and voting took place during a two-day workshop in December 2010 in Switzerland. Unfortunately, only 16 participants were able to attend. The remaining 400 questions assigned to the twelve topic areas (median: 33; range: 24–46/topic area) were discussed in four sessions. In each session, three groups worked simultaneously to reduce and rephrase the questions. In the plenary session the participants decided to reduce the questions to 50 instead of 55 due to overlaps. The final 50 questions were selected in a plenary session by majority vote following discussion.

### II) Classification and Analysis of the Questions

**Figure 2:**
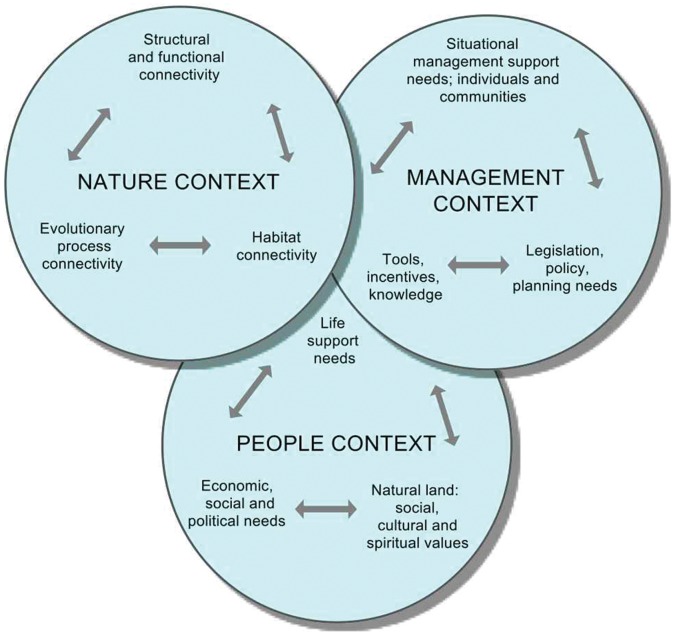
The three inter-related context areas of connectivity conservation adapted from Worboys et al. (2010). Every context area consists of three different sub-topics which interact with each other.

The core team ascribed individual questions from the final non-prioritized list of 50 questions, to three interacting context areas according to a slightly modified, previously published, management framework for connectivity conservation: nature-, people- and management contexts. Each context area has 3 interacting sub-topic areas, We subsequently assigned each question to one or more sub-topic area within the individual context areas (Figure 2) [15]. We selected the context area most likely concerned when addressing the question and the context area that would be affected by the outcome. This approach was given preference over linking questions to a context area with respect to the cause of the issue or question. For instance, question 5 “Which indicators reflect the changes in connectivity that result from climate or human induced changes in Alpine landscapes?” was assigned to “Structural and functional connectivity” and linked to “Tools, incentives, knowledge”, since the solution (“indicators” = tool, knowledge) provides answers to a particular problem (“changes in connectivity”). It is not a question concerning the causes of this problem (“climate or human induced changes”).

All questions were formulated either as (i) what - asking for information about something, (ii) which - asking about choice and (iii) how – questioning manner, condition or quality in order to further breakdown and classify the outcomes.

Finally, we established a simple non-prioritized list that attributes all 50 questions to the three context areas and respective sub-topics (Tables 1 and 2). The majority (54%) of the final 50 questions could be assigned to two context areas due to their transdisciplinary nature. Furthermore, we identified direct dependencies of individual questions on other questions, meaning that a specific question can only be solved if another question is answered beforehand. Finally, we created a graphic conceptualisation showing the interactions, inter-relations and dependencies of the questions.
